# Two Distinct Chronic Obstructive Pulmonary Disease (COPD) Phenotypes Are Associated with High Risk of Mortality

**DOI:** 10.1371/journal.pone.0051048

**Published:** 2012-12-07

**Authors:** Pierre-Régis Burgel, Jean-Louis Paillasseur, Bernard Peene, Daniel Dusser, Nicolas Roche, Johan Coolen, Thierry Troosters, Marc Decramer, Wim Janssens

**Affiliations:** 1 Service de Pneumologie, Hôpital Cochin, AP-HP and Université Paris Descartes, Sorbonne Paris Cité, Paris, France; 2 Clindatafirst, Clamart, France; 3 Respiratory Division, University Hospital Gasthuisberg, K.U. Leuven, Leuven, Belgium; 4 Service de Pneumologie, Hôpital de l’Hôtel Dieu, AP-HP and Université Paris Descartes, Sorbonne Paris Cité, Paris, France; 5 Radiology, University Hospital Gasthuisberg, K.U. Leuven, Leuven, Belgium; University at Buffalo, State University of New York, United States of America

## Abstract

**Rationale:**

In COPD patients, mortality risk is influenced by age, severity of respiratory disease, and comorbidities. With an unbiased statistical approach we sought to identify clusters of COPD patients and to examine their mortality risk.

**Methods:**

Stable COPD subjects (n = 527) were classified using hierarchical cluster analysis of clinical, functional and imaging data. The relevance of this classification was validated using prospective follow-up of mortality.

**Results:**

The most relevant patient classification was that based on three clusters (phenotypes). Phenotype 1 included subjects at very low risk of mortality, who had mild respiratory disease and low rates of comorbidities. Phenotype 2 and 3 were at high risk of mortality. Phenotype 2 included younger subjects with severe airflow limitation, emphysema and hyperinflation, low body mass index, and low rates of cardiovascular comorbidities. Phenotype 3 included older subjects with less severe respiratory disease, but higher rates of obesity and cardiovascular comorbidities. Mortality was associated with the severity of airflow limitation in Phenotype 2 but not in Phenotype 3 subjects, and subjects in Phenotype 2 died at younger age.

**Conclusions:**

We identified three COPD phenotypes, including two phenotypes with high risk of mortality. Subjects within these phenotypes may require different therapeutic interventions to improve their outcome.

## Introduction

Chronic obstructive pulmonary disease has long been categorized using the FEV_1_-based GOLD classification [1]. However, marked heterogeneity exists within each GOLD stage in terms of symptoms, exacerbations, quality of life and exercise capacity [2]. Mortality risk is also heterogeneous within each GOLD stage, because FEV_1_ is not the only determinant of mortality in COPD patients [3]. Other factors independently associated with survival include age, dyspnoea, health status, hyperinflation, gas exchange abnormalities, exacerbation frequency, exercise capacity, pulmonary hemodynamic, and nutritional status [4].

Recently, interest has emerged for the identification of clinical COPD phenotypes [5], as defined by “a single or combination of disease attributes that describe difference between individuals with COPD as they relate to clinically meaningful outcomes” [6]. Cluster analysis has appeared as a useful tool to identify subgroups of patients with airway diseases [7], [8], [9], [10], including subgroups of patients with COPD [11], [12].

In the present study, we performed a cluster analysis using multiple variables (including lung function, imaging, and comorbidities) obtained in a large cohort of COPD subjects recruited in stable condition. The clinical relevance of these clusters of subjects was validated using survival data obtained during longitudinal follow-up. Our aim was to examine whether clusters of COPD patients identified with an unsupervised approach differed in mortality.

## Methods

### Patients

**Figure 1:**
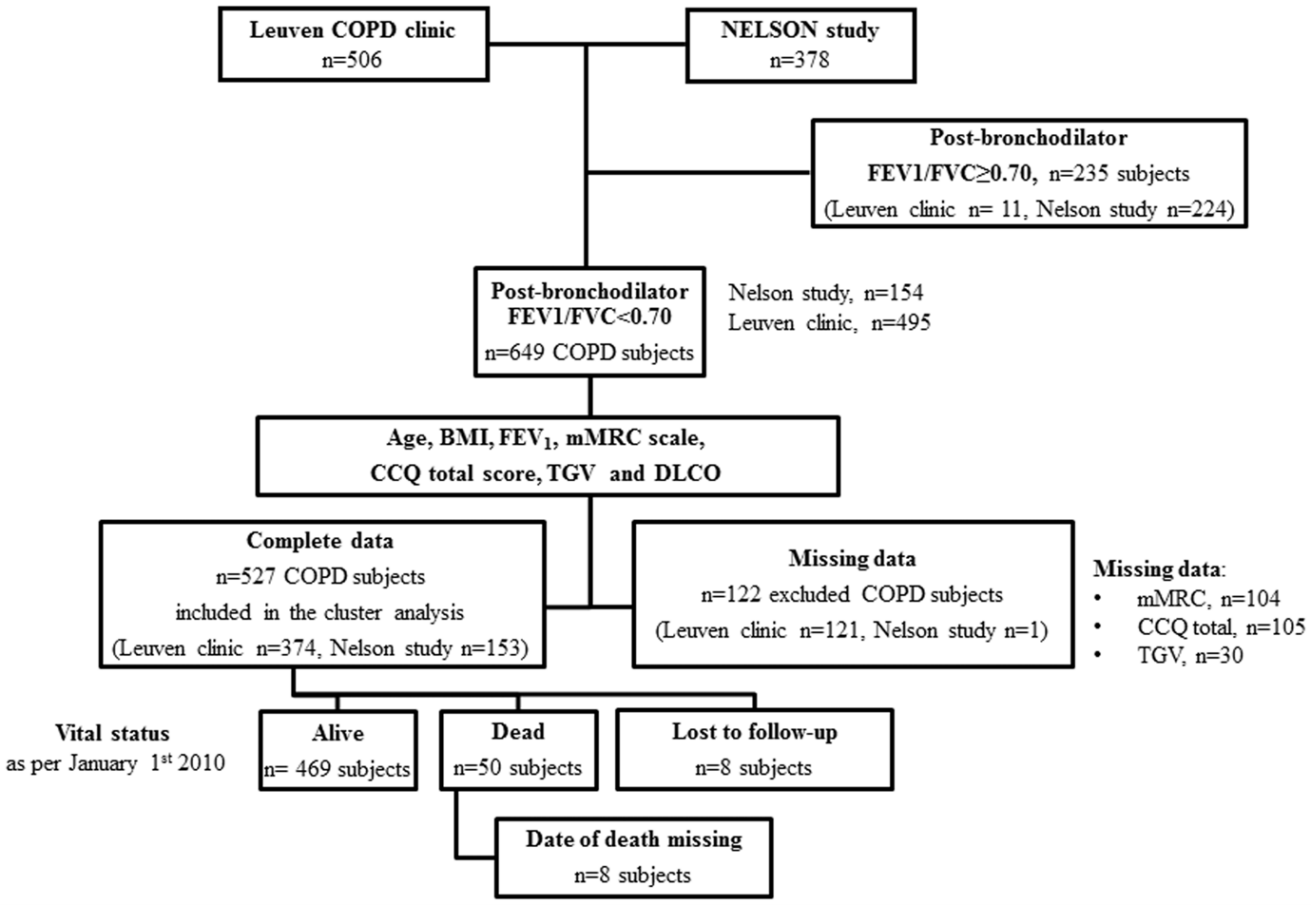
Flow chart. Abbreviations: BMI: body mass index; mMRC: modified Medical Research Council; CCQ: clinical COPD questionnaire; TGV: thoracic gas volume and DLCO: diffusing capacity of the lung for carbon monoxide.

Clinical, functional and imaging data obtained in COPD patients [1] at inclusion in the study (cross-sectional data) were analyzed using unsupervised analysis. Validation of the clinical relevance of these clusters of patients was achieved using survival data obtained during prospective follow-up. To ensure sufficient patient heterogeneity, subjects recruited in two separate cohorts were studied. The first cohort was composed of 506 subjects recruited at the LEUVEN university hospital COPD outpatient clinic. The second cohort was composed of 378 subjects recruited in the neighbourhood of LEUVEN as part of the Dutch-Belgian randomized lung cancer screening (NELSON study) [13]. Inclusion criteria in this latter cohort were a smoking history ≥15 pack-years and age >50 years, and only 154 patients had a diagnosis of COPD (according to a post-bronchodilator FEV_1_/FVC<0.70) [1]. Further, eleven patients were excluded from the cohort LEUVEN clinic cohort due to a FEV_1_/FVC ratio≥0.70. Thus, our COPD population was composed of 649 subjects (495 from the LEUVEN clinic and 154 from the NELSON study). The COPD subjects included in this cluster analysis were required to have complete information for 7 selected continuous variables (see below), leading to the exclusion of 122 COPD subjects (121 from the LEUVEN clinic) due to missing data. The final study population included in the cluster analysis contained 527 COPD (LEUVEN clinic n = 374; NELSON subjects, n = 153) [13]. A flow chart describing patient selection is provided in **Figure 1** **.** A description of characteristics of COPD patients recruited in the LEUVEN clinic and in the NELSON study and a description of the excluded COPD subjects is provided in **Table S2**. All studies were approved by the Ethics Committee at the University Hospitals of Leuven (Leuven, Belgium) and all participants provided written informed consent.

### Data Collection

Data were obtained at the time of inclusion in the studies. Demographic characteristics, post-bronchodilator pulmonary function assessment, CT scan of the chest, and questionnaires on dyspnoea (mMRC) and quality of life (CCQ) [14] were collected. In patients recruited at the LEUVEN clinic, data on comorbidities were obtained from medical records at the time of inclusion. Comorbidities of subjects enrolled via the NELSON study were obtained by detailed interview and review of concomitant medications at the time of inclusion. In case of doubt, general practitioners were contacted for double checking. Data on the following COPD-related comorbidities were collected: ischemic heart disease, stroke, peripheral arterial disease, diabetes, osteoporosis, skeletal muscle weakness (quadriceps force <80% predicted) and anaemia (haemoglobin <11 g/dl on last venous blood sample). Patients recruited in the NELSON study had no data available for peripheral arterial disease and muscle weakness.

The complete protocol used for CT imaging, which was based on National Emphysema Treatment Trial criteria [15], was described in a previous report [16]. Emphysema was semi-quantitatively assessed by a visual scoring system. Four categories were generated yielding a four-level alveolar destruction scale (no emphysema, mild emphysema affecting <20%, moderate emphysema between 20–50%, and severe emphysema >50% of the lung) [16]. Thickening of the bronchial walls was scored on a semi-quantitative three-level scale and presence or absence of bronchiectasis was assessed [16].

All pulmonary function data were obtained with standardized equipment (Jaeger) according to ATS/ERS consensus guidelines. Spirometric values were post-bronchodilator values. Diffusing capacity was determined by single breath carbon monoxide gas transfer method (DLCO) and corrected for alveolar ventilation (Kco) but not for haemoglobin. All data were obtained as absolute values and expressed as percent predicted of reference values [17].

### Plan for Cluster Analysis

Our strategy was to combine both continuous and categorical data in a single cluster analysis aimed at the identification of COPD phenotypes. Based on the result of a first analysis of this database (**Table S1**) we made a selection of continuous variables to be included the cluster analysis. This analysis resulted in the selection of 7 continuous variables (see below). Because some continuous variables were correlated with each other (**Table S3**), we eliminated correlations between variables by performing a principal component analysis. For categorical variables, all variables were used in the analysis but these variables were submitted to multiple correspondence analyses (MCA) to transform them into independent mathematical axes. This latter procedure allowed using in a single cluster analysis (Ward’s procedure) the significant axes identified in the MCA and the significant component identified by PCA. A description of these procedures is presented in **Text S1**.

### Processing of Continuous and Categorical Variables

Seven continuous variables were selected for their relevance to COPD natural history: age, body mass index (BMI), FEV_1_ (% predicted), mMRC scale, CCQ total score, thoracic gas volume (TGV, % predicted) and DLCO (% predicted). Subjects with complete data for these 7 variables were submitted to PCA. The first two axes identified in the PCA had eigenvalues >1 and were kept for cluster analysis (**Table S4 and S5**).

All categorical variables available were submitted to MCA. The variables included in these analyses were comorbidities, and data obtained from CT analysis, including emphysema, bronchial thickening and bronchiectasis. MCA identified 17 axes of which 3 were excluded because they happened to be correlated mostly with missing information on comorbidities (**Table S6 and S7)**. Thus, we were able to exclude these 3 axes without losing significant information and only 14 axes were kept for cluster analysis.

### Vital Status and Survival Analyses

Vital status was assessed as per January 1^st^ 2010. For patients followed at the University hospital, mortality data were obtained from medical files. When no data on mortality was retrieved, general practitioners (GP’s) caring for the patient were contacted to check survival. For subjects from the NELSON study, survival was checked by direct telephone contact with GP’s. Subjects who were lost to follow-up (n = 8) were not included in the survival analysis because no information was available on their vital status. Additionally the exact date of death was unavailable in 8 subjects who died during follow-up. Thus, the survival analyses were performed in 511/527 (97%) subjects.

Survival analyses were performed on all-cause mortality using Kaplan-Meier and log-rank tests with Tukey-Kramer adjustments for multiple comparisons. Because age was markedly different among Phenotypes, we further studied mortality risk using a Cox model adjusted for age.

### Statistics

Data are presented as median [interquartile range (IQR)] or %. A *P*<0.05 was considered statistically significant. Analyses were performed using the SAS 9.2 statistical software (Cary, North Carolina, USA).

## Results

### Characterization of COPD Patients Based on GOLD Classification

Characteristics of the 527 COPD patients according to the spirometric GOLD classification are presented in **Table 1**. Subjects recruited in the NELSON study were mostly in spirometric GOLD stage I and II (65% and 31%, respectively), whereas subjects recruited in the LEUVEN clinic were mostly in spirometric GOLD stage II, III and IV (33%, 38%, and 24%, respectively) (also see **Table S2)**. Increasing GOLD stage was associated with increased dyspnoea, decreased HRQoL (higher CCQ total score), a lower BMI, higher lung hyperinflation and decreased lung diffusing capacity. Extent of emphysema, bronchial thickening and bronchiectasis were also associated with more severe airflow limitation. Muscle weakness and osteoporosis increased with GOLD stages, whereas diabetes and cardiovascular comorbidities appeared relatively unrelated to the degree of airflow limitation.

**Table 1:** Description of the 527 COPD patients based on spirometric GOLD classification.

	GOLD I	GOLD II	GOLD III	GOLD IV
	n = 120	n = 169	n = 149	n = 89
**Demographic**				
**Age, yrs.**	62 [58–67]	68 [61–74]	68 [62–75]	61 [58–65]
**Male sex, %**	80	79	78	72
**BMI, kg/m^2^**	25 [24–28]	26 [23–28]	24 [20–27]	22 [19–25]
**Smoking, pack-year**	43 [32–55]	47 [34–61]	50 [32–64]	46 [33–60]
**Source of recruitment**				
NELSON study, % (% NELSON)	83 (65)	28 (31)	5 (4)	0 (0)
LEUVEN clinic, % (% LEUVEN)	17 (5)	72 (33)	95 (38)	100 (24)
**Pulmonary function tests**				
**FEV_1_, % predicted**	93 [87–103]	64 [57–71]	40 [36–44]	24 [20–28]
**FEV_1_, L**	2.9 [2.5–3.2]	1.8 [1.5–2.1]	1.1 [0.9–1.3]	0.7 [0.6–0.8]
**FVC, % predicted**	115 [106–126]	94 [85–105]	79 [70–89]	64 [54–74]
**FVC, L**	4.5 [3.8–5.0]	3.3 [2.8–4.1]	2.8 [2.4–3.3]	2.2 [1.7–2.9]
**FEV_1_/FVC ratio**	0.66 [0.63–0.68]	0.55 [0.48–0.60]	0.39 [0.35–0.44]	0.31 [0.25–0.35]
**RV, % predicted**	115 [101–133]	132 [109–155]	171.0 [143–199]	227 [181–271]
**TLC, % predicted**	109 [102–117]	104 [93–114]	112 [101–121]	124 [110–136]
**TGV, % predicted**	117 [107–133]	130 [110–151]	161 [137–177]	193 [169–217]
**Raw, % predicted**	152 [126–187]	189 [164–240]	257 [224–318]	355 [274–427]
**Sgaw, % predicted**	82 [67–99]	61 [48–75]	36 [31–46]	25 [21–31]
**DLCO, % predicted**	80 [66–91]	58 [49–74]	45 [34–57]	33 [27–38]
**Kco, % predicted**	86 [73–98]	79 [63–92]	64 [52–87]	56 [45–73]
**Symptoms**				
**Dyspnoea, mMRC scale**	0 [0–1]	1 [0–2]	2 [1]–[3]	3 [1]–[3]
**Clinical COPD Questionnaire, Total score**	1.8 [0.8–3.5]	3.5 [1.8–6.3]	5.5 [3.5–7.8]	6.8 [5.3–9.0]
**Comorbidities**				
**Ischemic heart disease, %**	14	27	23	26
**Stroke, %**	2.5	3	4	6
**Peripheral artery disease, %** *	14*	21*	12	11
**Diabetes, %**	8	17	14	13
**Muscle weakness, %** *	5*	29*	40	58
**Osteoporosis, %**	5	15	17	39
**Anaemia,** %	6	7	12	9
**CT scan**				
**Emphysema present, %**	39	69	82	92
**Alveolar destruction**				
Absent, %	61	31	18	8
Mild, %	31	38	26	13
Moderate, %	7	22	29	30
Severe, %	1	9	26	49
**Bronchial thickening**				
Mild, %	64	37	24	32
Moderate, %	30	45	49	48
Severe, %	6	18	27	20
**Bronchiectasis**, %	12	26	29	32
**Mortality**				
**Deaths, n (%)**	1 (0.8)	5 (3.0)	21 (14.1)	23 (25.8)

BMI : body mass index; FEV1: forced expiratory volume in 1 sec, FVC: forced vital capacity, RV: residual volume, TLC: total lung capacity, TGV: thoracic gas volume, Raw: airway resistance, Sgaw: specific airway conductance, DLCO: diffusing capacity of the lung for carbon monoxide, KCO: ratio of DLCO to alveolar volume, mMRC: modified Medical Research Council Scale.

* , % missing data: GOLD I 83%, GOLD II 28%.

The median follow-up duration was of 17.2 [10.8; 22.9] months, and was not different among GOLD stages (*not shown*). At that time, 8/527 (1.5%) patients were lost to follow-up and 50/519 (9.6%) subjects had died. Mortality rates were 0.8%, 3.0%, 14.1% and 25.8% in spirometric GOLD stage I, II, III and IV subjects, respectively.

### Identification of COPD Phenotypes using Cluster Analysis and Mortality Rates

**Figure 2:**
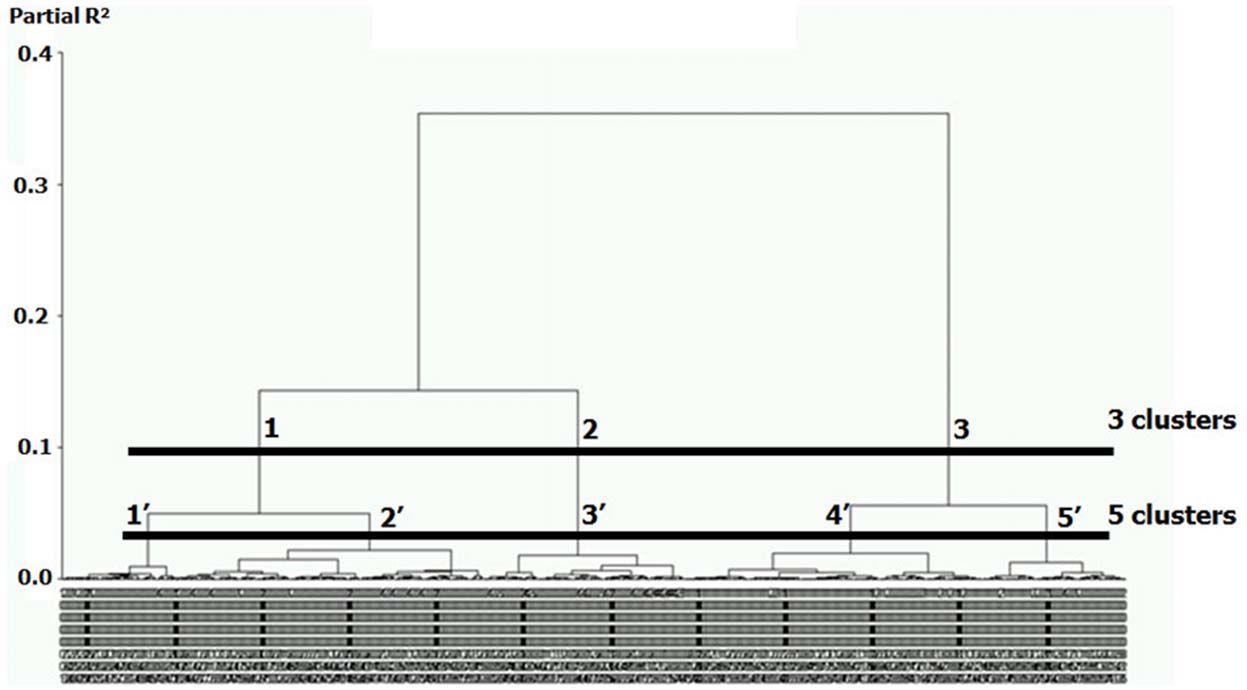
Dendrogram illustrating the results of the cluster analysis in 527 COPD subjects. Subjects were classified using agglomerative hierarchical cluster analysis based on the main axes identified by principal component analysis (PCA) and multiple correspondence analyses (MCA, see Methods section). Each vertical line represents an individual subject and the length of vertical lines represents the degree of similarity between subjects. The horizontal lines identify possible cut-off for choosing the optimal number of clusters in the data. When choosing 3 clusters (upper line) the 3 groups (labelled 1 to 3) have differential mortality rates (0.5%, 20.6% and 14.3% for Phenotype 1, 2, and 3, respectively). When choosing 5 clusters (lower line, labelled 1′ to 5′), subjects in clusters 1′ and 2′ had comparable mortality rates (0.7% and 0%, respectively) and subjects in clusters 4′ and 5′ had similar mortality rates (14.3% in each group), suggesting that grouping in 5 phenotypes would not improve patient classification.

We performed a Ward’s cluster analysis based on the significant mathematical axes identified by PCA and MCA for continuous and categorical variables, respectively. Classification of the 527 COPD patients resulted in a dendrogram showing the progressive joining of the clustering process (**Figure 2**). Based on visual assessment of the dendrogram, data could be optimally grouped into 3 or 5 clusters, each cluster corresponding to a potential phenotype. To decide on the number of phenotypes, we examined mortality rates among clusters. When grouping the data into 3 clusters, there was a clear difference in mortality rates among clusters (**Table 2** **and** **Figure 2**). Grouping the data into 5 clusters did not improve the ability to predict mortality because this only resulted in the division of clusters 1 and 3 into two new clusters (for each), but mortality was comparable in these newly formed clusters (**Figure 2**).

**Table 2:** Description of the 527 COPD patients based on phenotypes identified by cluster analysis.

	Phenotype 1	Phenotype 2	Phenotype 3	*P* value
	n = 219	n = 99	n = 209	
**DATA USED IN THE CLUSTER ANALYSIS**				
**Quantitative data**				
**Age, yrs.**	62 [58–68]	61 [57–66]	72 [65–77]	<0.001
**BMI, kg/m^2^**	25 [23–28]	20 [18–22]	26 [24–29]	<0.001
**FEV_1_, % predicted**	80 [65–94]	29 [21–37]	44 [36–58]	<0.001
**Dyspnoea, mMRC scale**	0 [0–1]	2 [1]–[3]	2 [2]–[3]	<0.001
**Clinical COPD Questionnaire, Total**	1.8 [1.0–3.0]	6.8 [5.0–9.0]	6.3 [4.5–8.0]	<0.001
**TGV, % predicted**	126 [112–147]	195 [177–220]	139 [115–161]	<0.001
**DLCO, % predicted**	74 [60–86]	34 [25–39]	47 [38–61]	<0.001
**Categorical data**				
**CT scan** *				
**Emphysema present, %**	48	96	81	<0.001
**Alveolar destruction**				
Absent (%)	52	4	19	<0.001
Mild (%)	35	9	32	
Moderate (%)	8	43	27	
Severe (%)	5	44	22	
**Bronchial thickening**				
Mild (%)	57	36	20	<0.001
Moderate (%)	33	45	52	
Severe (%)	9	19	27	
**Bronchiectasis, %**	14	31	33	<0.001
**Comorbidities**				
**Ischemic heart disease, %**	14	17	34	<0.001
**Stroke, %**	0	2	8	<0.001
**Peripheral arterial disease** * **, %**	10	11	19	0.08
**Diabetes, %**	9	11	20	0.003
**Muscle weakness** * **, %**	18	47	42	<0.001
**Osteoporosis, %**	8	31	22	<0.001
**Anaemia, %**	5	15	10	0.005
**DATA NOT USED IN THE CLUSTER ANALYSIS**				
**Male sex, %**	80	66	80	0.007
**GOLD stage**				<0.001
G I, % (% of all GOLD stage I)	50 (91)	0 (0)	5 (9)	
G II,% (% of all GOLD stage II)	39 (51)	8 (5)	36 (44)	
G III, % (% of all GOLD stage III)	9 (13)	37 (25)	44 (62)	
G IV, % (% of all GOLD stage IV)	2 (5)	55 (61)	15 (35)	
**Source of recruitment**				
NELSON cohort, % (% NELSON)	67 (95)	0 (0)	3 (5)	<0.001
LEUVEN clinic, % (% LEUVEN)	33 (19)	100 (27)	97 (54)	<0.001
**Smoking, pack-year**	44 [34–60]	48 [33–56]	48 [31–65]	0.89
**FEV_1_, L**	2.4 [1.9–2.9]	0.8 [0.6–1.0]	1.25 [1.0–1.6]	<0.001
**FVC, % predicted**	105 [92–118]	72 [61–86]	82 [69–94]	<0.001
**FVC, L**	4.0 [3.3–4.6]	2.6 [1.9–3.2]	2.8 [2.3–3.3]	<0.001
**FEV_1_/FVC ratio**	0.62 [0.53–0.67]	0.33 [0.27–0.38]	0.44 [0.37–0.55]	<0.001
**RV,% predicted**	128 [109–149]	227 [186–268]	144 [113–174]	<0.001
**TLC, % predicted**	106 100–118]	128 [116–139]	104 [91–114]	<0.001
**Raw, % predicted**	174 [140–223]	274 [215–379]	249 [198–317]	<0.001
**Sgaw, % predicted**	68 [51–87]	29 [22–40]	44 [32–64]	<0.001
**Kco, % predicted**	86 [71–98]	53 [45–62]	70 [56–91]	<0.001
**Mortality, n (%)**	1 (0.5%)	20 (20.6%)	29 (14.3%)	<0.001

* % missing data: Phenotype 1∶67%; Phenotype 2∶1%, Phenotype 3∶4%.

P values correspond to comparisons between the 3 phenotypes using Kruskal-Wallis or Chi-square tests, as appropriate.

### Characterization of COPD Phenotypes

Characteristics of subjects grouped into 3 clusters (phenotypes) are presented in **Table 2**.

Phenotype 1 (n = 219 subjects) corresponded to subjects with a median [IQR] age of 62 [58–68] yrs., mild to moderate airflow limitation, absent or mild emphysema, absent or mild dyspnoea, normal nutritional status and limited comorbidities. Two third of these subjects were recruited in the NELSON study whereas one third of these subjects were recruited in the LEUVEN clinic. Of note, 95% of the NELSON subjects clustered in this phenotype. Only 1/219 (0.5%) subject died in this phenotype.

Phenotype 2 (n = 99 subjects) corresponded to subjects with a median [IQR] age of 61 [57–66] yrs., severe airflow limitation, marked emphysema and hyperinflation, low BMI, severe dyspnoea, and impaired HRQoL. One third of these subjects were women, and osteoporosis and muscle weakness were highly prevalent, whereas diabetes and cardiovascular comorbidities were less prevalent. Two subjects were lost to follow-up and mortality rates were very high with 20/97 (20.6%) deaths.

Phenotype 3 (n = 209 subjects) mostly corresponded to male subjects with a median [IQR] age of 72 [65–77] yrs., and moderate to severe airflow limitation. These subjects had less severe emphysema than subjects in Phenotype 2, but higher prevalence of bronchial thickening. They were often obese and had high rates of diabetes and cardiovascular comorbidities. Six subjects were lost to follow-up and mortality rates were also high with 29/203 (14.3%) deaths.

### Survival Pattern According to Phenotypes

Median [IQR] follow-up times were 2.4 [1.8; 2.9] yrs. for Phenotype 1, 2.3 [1.8; 2.8] yrs. for Phenotype 2, and 2.5 [2.1; 2.9] yrs. for Phenotype 3 and were not significantly different (*P* = 0.13; Kruskal-Wallis test).

**Figure 3:**
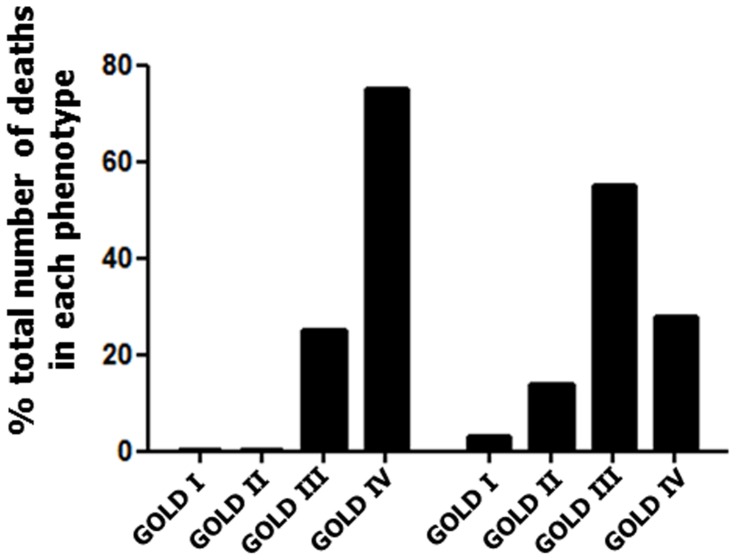
Mortality distribution by GOLD stage in Phenotype 2 and 3. At the end of the follow-up period, 20/97 (20.6%) and 29/203 (14.3%) subjects had died in Phenotype 2 and 3, respectively. Distribution of dead subjects by GOLD stage is expressed as % total number of death in each phenotype. The majority of Phenotype 2 subjects who died had very severe airflow limitation, whereas only 25% of Phenotype 3 subjects who died were in GOLD stage IV.

When comparing Phenotypes 2 and 3, in which subjects were at high risk of mortality, the pattern of mortality was different. In Phenotype 2, 75% of subjects who died were in GOLD stage IV and 25% were in GOLD stage III, indicating that the mortality pattern followed the severity of airflow obstruction. By contrast, in Phenotype 3, mortality distributed among all GOLD stages (**Figure 3**).

**Figure 4:**
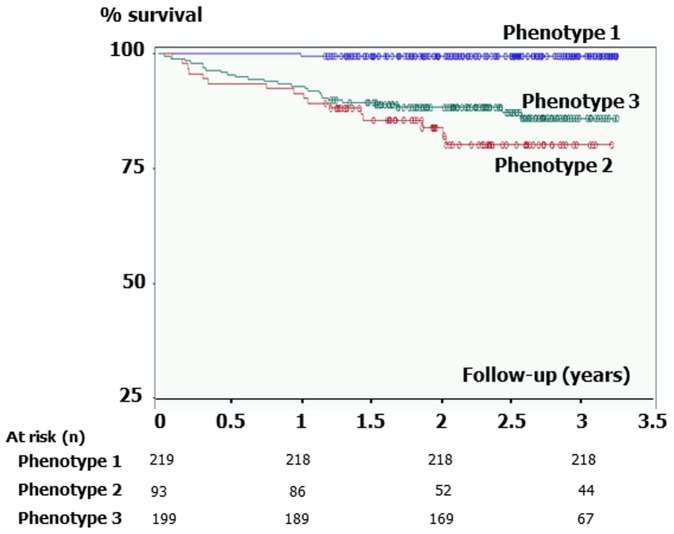
Kaplan-Meier analysis of mortality between Phenotypes. Subjects in Phenotype 2 and 3 were at higher risk of mortality than subjects in Phenotype 1 (each comparison, *P*<0.0001; log-rank test). However, no significant difference was observed between Phenotype 2 and 3, indicating that during the period of observation both group had comparable mortality.

Kaplan-Meier analysis of mortality between the 3 phenotypes is presented in **Figure 4**. Subjects in Phenotype 2 and 3 were at higher risk of mortality than subjects in Phenotype 1 (each comparison, *P*<0.0001; log-rank test), but no significant difference was observed between Phenotype 2 and 3. Because age at inclusion was markedly different between these latter phenotypes (median age, 61 yrs. vs. 72 yrs.), we hypothesized that subjects in Phenotype 2 had died earlier in life than subjects in Phenotype 3. Median [IQR] age of death was 64.5 [60.4–68.9] yrs. in Phenotype 2 (n = 16) and was 75.9 [70.8–77.8] yrs. in Phenotype 3 (n = 25). To take this difference into account, we performed Cox model analyses of mortality using phenotypes and age as covariates (**Table 3** **).** After adjustment for age, subjects in Phenotype 2 had a 3-fold increase in mortality compared with subjects in Phenotype 3.

**Table 3:** Cox model analysis of mortality between phenotypes.

	Unadjusted		Adjusted for age	
	Hazard Ratio [95% CI]	*P* value	Hazard Ratio [95% CI]	*P* value
*Phenotype 2 vs. 3*	1.4 [0.8;2.7]	0.23	3.3 [1.5; 7.2]	0.002
*Phenotype 2 vs. 1*	42.4 [5.6; 320.1]	0.0003	47.5 [6.3; 358.6]	0.0002
*Phenotype 3 vs. 1*	28.9 [3.9;213.3]	0.001	14.3 [1.9; 110;3]	0.01

CI: confidence interval.

## Discussion

In this large population of COPD subjects with a wide range of airflow limitation, we identified three COPD phenotypes, including one phenotype at low risk of mortality and two distinct phenotypes (Phenotype 2 and 3) at high risk of mortality. Phenotype 2 included younger patients with severe respiratory disease, low BMI and low rates of cardiovascular comorbidities. Phenotype 3 included older patients with less severe airflow limitation, but who were often obese and had higher rates of cardiovascular comorbidities and diabetes. These findings suggest that different strategies for improving outcome should be proposed to these two groups of COPD patients.

We have identified clusters of COPD subjects, which were associated with different mortality rates and patterns, qualifying as phenotypes [6]. In a French cohort of COPD subjects, investigators identified four clusters of subjects, including two clusters of subjects at high risk of predicted mortality [11]. In the present study, the two phenotypes that were at high risk of actual mortality were very similar to those identified in the French study [11]. Because all subjects had extensive characterization, including complete lung function assessment and CT scan, our current data further improve the description of these phenotypes. Garcia-Aymerich et al. also performed a cluster analysis of 342 Spanish subjects hospitalized for the first time because of COPD exacerbation [12]. The authors described 3 phenotypes, including “a severe respiratory phenotype” and “a systemic COPD phenotype” [12], which were at high risk of serious events (hospitalization and/or mortality). The “systemic COPD phenotype” was characterized by high prevalence of obesity and cardiovascular disease [12], corresponding to our Phenotype 3. However, the “severe respiratory phenotype” differed from our Phenotype 2 in that patients were not younger and had no malnutrition [12]. Such difference may be related to the recruitment of a specific population of subject at the time of first hospitalization for COPD exacerbation. Female represented only 6–8% of subjects in the Garcia-Aymerich’s study, whereas they represented up to one third of subjects in our Phenotype 2. Interestingly, recent data suggested that female gender is a risk factor for early onset COPD and more severe disease at young age [18]. These phenotypic differences underline the need for external validation of identified phenotypes across multiple populations.

Some limitations have to be taken into account when interpreting our results. Although repeated and severe exacerbations are important predictors of mortality [19], we had no data on exacerbations. Our study was based on the assessment of COPD patients coming to an outpatient clinic and smokers recruited for a study on lung cancer screening. Although these patients had a wide range of disease severity, they may not represent the COPD population at large and different results may be observed when studying different populations of patients. COPD subjects recruited as part of the NELSON study [13] were submitted to systematic screening and may not be representative of symptomatic subjects receiving a diagnosis of COPD. The inclusion of these subjects allowed for studying COPD subjects with a wide range of disease severity because the NELSON subjects were mostly in GOLD stage I and II, whereas the LEUVEN subjects were mostly in GOLD stage II, III and IV. Interestingly, 95% of the NELSON subjects and 19% of the LEUVEN subjects clustered in Phenotype 1, in which mortality was almost absent. Thus, our methodology was able to identify subjects at low risk of mortality in subjects with previously diagnosed and with previously undiagnosed COPD. In this real-life COPD population, 8/527 (1.5%) subjects were lost to follow-up and the exact date of death was unavailable in 8/50 (16%) subjects who died during follow-up. Because survival analyses were performed in 511/527 (97%) subjects, missing data were unlikely to significantly affect our results. Our survival analyses were based on all-cause mortality and specific causes of mortality could not be determined, which prevented us from determining whether causes of death differed between phenotypes. Phenotype 2 subjects who died during follow-up were mostly in GOLD stage IV, whereas Phenotype 3 subjects who died distributed in all GOLD stages (**Figure 3**), suggesting that airflow obstruction was not its main determinant. Although it is likely that subjects in Phenotype 2 had higher rates of lung function decline, further studies specifically assessing lung function decline with longitudinal spirometric data will be required to confirm this hypothesis. Assessment of comorbidities was based on physician-diagnosed comorbidities and not on a systematic diagnostic work-up. Because high rates of underdiagnosed cardiovascular comorbidities have been previously reported in COPD patients [20], we cannot exclude that such comorbidities contributed to death in some patients without any diagnosed concomitant disease. Finally, our methodology lead to the exclusion of 122 COPD patients who had missing data for mMRC and CCQ scores (see Methods). A careful investigation of this group of patients revealed that 107/122 patients were included at the very beginning of the LEUVEN outpatient cohort and belonged to a group of patients with severe airflow limitation (median [IQR] FEV_1_ 24 [19–31]) at a median [IQR] age of 57 [54–62] yrs. who had few comorbidities and were evaluated for lung transplantation (**Table S8**). Although we were unable to include the patients in our analysis, these findings further reinforce our conclusions, as the excluded patients could correspond to subjects in Phenotype 2.

Although some therapies (e.g., smoking cessation, pulmonary rehabilitation, bronchodilators) may be beneficial in all COPD subjects, differential characteristics of subjects in Phenotype 2 and Phenotype 3 suggest that different strategies may be developed for improving outcome, eventually resulting in better survival. Subjects in Phenotype 2 may preferentially benefit from lung transplantation as they are younger and have little co-morbidities. Early detection of subjects in Phenotype 2 would allow for early intervention, with the goal of developing disease-modifying therapy. Thus, future treatment targeting airway and parenchymal disease progression (e.g., growth factor receptor antagonists, protease inhibitors) may be of particular interest in these subjects with severe and early onset respiratory disease. We also speculate that interventions (e.g., aspirin, statins, beta-blockers) shown to reduce mortality in subjects with cardiovascular diseases may show optimal survival benefit in older subjects with cardiovascular comorbidities (Phenotype 3).

In summary, this study identified two very different phenotypes of subjects at high risk of mortality: younger subjects with severe respiratory disease and emphysema, and older subjects with less severe respiratory disease and marked cardiovascular and metabolic comorbidities. Pathophysiological studies should take these phenotypes into account to determine whether they relate to specific mechanisms and/or are differentially associated with specific genotypic signature or biomarkers. Further, potential therapeutic implication of these phenotypes can now be examined in prospective trials. Future studies should also focus on establishing simple algorithms based on the most discriminant factors for assigning patients to specific phenotypes. Such algorithms will have to be tested in validation cohorts before they can be utilized in clinical practice.

## Supporting Information

Text S1 **Additional information on statistical analyses.**
(DOC)

Table S1 **Cluster analysis showing the relationships between continuous variables in 519 COPD subjects.**
(DOC)

Table S2 **Main characteristics of the 527 COPD subjects included in the cluster analysis, according to their cohort of recruitment (Leuven outpatient clinic and NELSON study).**
(DOC)

Table S3 **Correlation matrix between variables used in the cluster analysis.**
(DOC)

Table S4 **Eigenvalues of the correlation matrix.**
(DOC)

Table S5 **Principal component analysis of 7 continuous variables in 527 patients: correlation coefficients between variables and components identified by principal component analysis.**
(DOC)

Table S6 **Relative contribution of the 17 dimensions identified in the multiple correspondence analyses.**
(DOC)

Table S7 **Correlations of the original categorical variables with the 17 dimensions derived from the multiple correspondence analyses.**
(DOC)

Table S8 **Comparison of included vs. excluded subjects from the cluster analysis.**
(DOC)
