# Supplementary material for: Two Distinct Chronic Obstructive Pulmonary Disease (COPD) Phenotypes Are Associated with High Risk of Mortality
Source: PLoS One. 2012 Dec 7;7(12):e51048. doi: 10.1371/journal.pone.0051048 (PMC3517611; doi:10.1371/journal.pone.0051048)
Supplement: Text S1 — Additional information on statistical analyses. (DOC) [file pone.0051048.s001.doc]

**Text S1**

**Title: Two distinct COPD phenotypes are associated with high risk of mortality**

**Authors**: Pierre-Régis Burgel, Jean-Louis Paillasseur, Bernard Peene, Daniel Dusser, Nicolas Roche, Johan Coolen, Thierry Troosters, Marc Decramer and Wim Janssens

**Processing of continuous variables**

The COPD population contained 649 COPD subjects (see main manuscript), as defined by a post BD FEV_1_/FVC<70%. First the relationship between continuous data that were present in the database was examined by performing a cluster analysis using the VARCLUS procedure. The results of this analysis are summarized in **Table S1** obtained in the 519 COPD patients with complete data for all variables listed in the first column.

Based on this classification of variables in four clusters, variables to be used in the principal component based-cluster analysis for the search of COPD phenotypes were selected. The choice was somewhat arbitrary: it was decided to keep in the model data that seemed clinically important for COPD; variables that were highly correlated with one another were excluded (e.g., Kco was excluded because DLCO was chosen) and at least one variable from each of the four clusters was kept. This resulted in the choice of the following 7 variables: age, BMI, FEV_1_ (% pred), mMRC, CCQ Total, TGV (% pred) and DLCO (% pred). All the subsequent analyses were performed on the COPD patients with complete data for these 7 variables (n=527). The main characteristics of these 527 subjects according to their cohort of recruitment (LEUVEN clinic vs. NELSON study) are presented in **Table S2**. Next, we examined the correlation of the 7 selected variables with each other in these 527 patients: the correlation matrix is given in supplementary **Table S3**. As shown in **Table S3**, some of the variables were highly correlated (for example, FEV_1_ was correlated with DLCO and inversely correlated with TGV). A principal component analysis was performed on these 7 variables, allowing for eliminating correlations between variables. Linear combinations of the seven selected variables were used to form seven new independent variables (also called axes or components). The eigenvalue of each component is a measure of its variability. A component with an eigenvalue <1 contributes little to explain the relationship between original variables and thus is not subjected to further analysis. The eigenvalues of the correlation matrix are given in **Table S4**. The first two components, which accounted for 65% of the information, had eigenvalues >1 and were kept for the subsequent cluster analysis. **Table S5** show the correlations of the seven original variables with the 7 components (Comp) derived from the principal component analysis.

**Processing of categorical variables**

The variables that were included in this analysis were (i) comorbidities (diabetes, ischemic heart disease, stroke, peripheral arterial disease, muscle weakness, osteoporosis, anemia), which could be classified as present, absent or missing information and (ii) data obtained from CT analysis, including emphysema (present or absent), alveolar destruction (4 classes), bronchial thickening (3 classes) and bronchiectasis (present or absent)**.** The multiple correspondence analyses resulted in the identification of 17 axes. The relative contribution of these 17 axes to the variability of information is presented in **Table S6**. **Table S7** shows the show the correlations of the original variables with the 17 dimensions derived from the MCA.

**Selection of the MCA axes**

Dimension 1 carries missing information for some comorbidities (diabetes, stroke, osteoporosis, anemia) with a column sum= 93%. Dimensions 2 and 17 carry information for missing variables from CT scan analysis (column sum for Dim 2=75.6% and for Dim 17=93%). These 3 dimensions contribute for 33.9 % of the variability of the information (see Table 5). Thus the removal of these 3 axes allows keeping 66.1% of the variability of the information in all the 527 patients.

**Cluster analysis for identification of Phenotypes**

A single cluster analysis (Ward’s procedure) was performed using a combination of the significant components identified by Principal Component Analysis of continuous variables (n=2 components or axes), and the 14 axes obtained by MCA of categorical variables.

**Comparison of subjects included vs. excluded from the cluster analysis**

As explained in the Method section of the main manuscript, 122 patients were excluded from our main analysis because they no data for mMRC and/or CCQ and/or TGV. A comparison of the characteristics of included vs. excluded patients is provided in **Table S8**. The 122 excluded patients were relatively younger but had very severe airflow limitation. These patients were included at the very beginning of our cohort (explaining longer follow-up time) and we found that 107/122 patients were evaluated for lung transplantation. Importantly most of these patients underwent lung transplant before 2010, preventing us for studying survival accurately in this group of patients (because survival would then depend on COPD severity, time of lung transplantation, and outcome of transplantation). Interestingly, these patients would correspond to the patients identified in our Phenotype 2 (young patients with severe respiratory disease and limited comorbidities), further validating the concept that younger patients with severe and predominant respiratory disease should be considered for developing specific therapies.
