# Supplementary material for: Two Distinct Chronic Obstructive Pulmonary Disease (COPD) Phenotypes Are Associated with High Risk of Mortality
Source: PLoS One. 2012 Dec 7;7(12):e51048. doi: 10.1371/journal.pone.0051048 (PMC3517611; doi:10.1371/journal.pone.0051048)
Supplement: Table S1 — Cluster analysis showing the relationships between continuous variables in 519 COPD subjects. (DOC) [file pone.0051048.s002.doc]

| **4 Clusters** | | **R-squared with** | | **1-R^2^ Ratio** |
| --- | --- | --- | --- | --- |
| **Cluster** | **Variable** | **Own Cluster** | **Next Closest** |  |
| **Cluster 1** | **FEV_1_, % pred** | 0.8549 | 0.4448 | 0.2613 |
|  | **FVC, % pred** | 0.8164 | 0.1961 | 0.2283 |
|  | **SVC, % pred** | 0.8102 | 0.1645 | 0.2272 |
|  | **Raw, % pred** | 0.6508 | 0.1700 | 0.4207 |
|  | **Sgaw, % pred** | 0.6858 | 0.3413 | 0.4770 |
| **Cluster 2** | **BMI** | 0.3342 | 0.0917 | 0.7331 |
|  | **RV, % pred** | 0.8627 | 0.3942 | 0.2267 |
|  | **TLC, % pred** | 0.8080 | 0.0513 | 0.2024 |
|  | **TGV, % pred** | 0.9398 | 0.2370 | 0.0790 |
| **Cluster 3** | **mMRC** | 0.6422 | 0.2938 | 0.5066 |
|  | **CCQ total** | 0.6069 | 0.2203 | 0.5042 |
|  | **DLCO, % pred** | 0.8129 | 0.3576 | 0.2913 |
|  | **KCO, % pred** | 0.6193 | 0.2360 | 0.4982 |
| **Cluster 4** | **Age** | 0.5375 | 0.0411 | 0.4823 |
|  | **Pack-years** | 0.5375 | 0.0087 | 0.4665 |

FEV_1_: forced expiratory volume in 1 sec, FVC : forced vital capacity; SVC: slow vital capacity, Raw: airway resistance, Sgaw: specific airway conductance, BMI : body mass index, RV: residual volume, TLC: total lung capacity, TGV: thoracic gas volume, mMRC: modified Medical Research Council Scale. CCQ total: clinical COPD questionnaire total score. DLCO: diffusing capacity of the lung for carbon monoxide; KCO: carbon monoxide transfer coefficient.
