# Supplementary material for: Two Distinct Chronic Obstructive Pulmonary Disease (COPD) Phenotypes Are Associated with High Risk of Mortality
Source: PLoS One. 2012 Dec 7;7(12):e51048. doi: 10.1371/journal.pone.0051048 (PMC3517611; doi:10.1371/journal.pone.0051048)
Supplement: Table S2 — Main characteristics of the 527 COPD subjects included in the cluster analysis, according to their cohort of recruitment (Leuven outpatient clinic and NELSON study). (DOC) [file pone.0051048.s003.doc]

|  | **LEUVEN** | **NELSON** |
| --- | --- | --- |
|  | **n=374 subjects** | **n=153 subjects** |
| **Age, yr** | 66 [61 – 74] | 62 [58 – 68] |
| **Male sex, %** | 76 | 82 |
| **BMI** | 24 [20 – 27] | 25 [23 – 28] |
| **Smoking, pack-year** | 48 [33 – 63] | 43 [34 – 56] |
| **FEV_1_, % predicted** | 42 [32 – 58] | 88 [73 – 99] |
| **FEV_1_, L** | 1.2 [0.8 – 1.6] | 2.6 [2.1 – 3.1] |
| **FVC, % predicted** | 82 [69 – 96] | 108 [97 – 122] |
| **FVC, L** | 2.9 [2.3 – 3.5] | 4.1 [3.5 – 4.8] |
| **GOLD stage** |  |  |
| G I, % | 5 | 65 |
| G II,% | 33 | 31 |
| G III, % | 38 | 4 |
| G IV, % | 24 | 0 |
| **Dyspnea, mMRC scale** | 2 [1 – 3] | 0 [0 – 1] |
| **Clinical COPD Questionnaire,**  **Total score** | 5.8 [3.8 – 8.0] | 1.5 [0.75 – 2.5] |
| **Comorbidities** |  |  |
| Ischemic heart disease, % | 27 | 12 |
| Stroke, % | 5 | 0 |
| Peripheral artery disease, %* | 15 | N/A |
| Diabetes, % | 16 | 9 |
| Muscle weakness, %* | 39 | N/A |
| Osteoporosis, % | 22 | 7 |
| Anaemia, % | 10 | 5 |

N/A: not available
