# Supplementary material for: Two Distinct Chronic Obstructive Pulmonary Disease (COPD) Phenotypes Are Associated with High Risk of Mortality
Source: PLoS One. 2012 Dec 7;7(12):e51048. doi: 10.1371/journal.pone.0051048 (PMC3517611; doi:10.1371/journal.pone.0051048)
Supplement: Table S3 — Correlation matrix between variables used in the cluster analysis. (DOC) [file pone.0051048.s004.doc]

|  | **Age** | **BMI** | **FEV1 % pred** | **mMRC** | **CCQ total** | **TGV % pred** | **DLCO % pred** |
| --- | --- | --- | --- | --- | --- | --- | --- |
| **Age** | 1.0000 | 0.0577 | -.0810 | 0.1796 | 0.0277 | -.1634 | -.0699 |
| **BMI** | 0.0577 | 1.0000 | 0.2234 | -.0805 | -.1167 | -.4517 | 0.3800 |
| **FEV_1_ % pred** | -.0810 | 0.2234 | 1.0000 | -.5686 | -.5206 | -.5855 | 0.7148 |
| **mMRC** | 0.1796 | -.0805 | -.5686 | 1.0000 | 0.6833 | 0.2840 | -.5757 |
| **CCQ total** | 0.0277 | -.1167 | -.5206 | 0.6833 | 1.0000 | 0.3185 | -.5257 |
| **TGV %pred** | -.1634 | -.4517 | -.5855 | 0.2840 | 0.3185 | 1.0000 | -.4861 |
| **DLCO % pred** | -.0699 | 0.3800 | 0.7148 | -.5757 | -.5257 | -.4861 | 1.0000 |
