# Supplementary material for: Two Distinct Chronic Obstructive Pulmonary Disease (COPD) Phenotypes Are Associated with High Risk of Mortality
Source: PLoS One. 2012 Dec 7;7(12):e51048. doi: 10.1371/journal.pone.0051048 (PMC3517611; doi:10.1371/journal.pone.0051048)
Supplement: Table S4 — Eigenvalues of the correlation matrix. (DOC) [file pone.0051048.s005.doc]

| **Eigenvalues of the Correlation Matrix** | | | | |
| --- | --- | --- | --- | --- |
|  | **Eigenvalue** | **Difference** | **Proportion** | **Cumulative** |
| **Comp 1** | 3.26602631 | 1.95251910 | 0.4666 | 0.4666 |
| **Comp 2** | 1.31350721 | 0.40104182 | 0.1876 | 0.6542 |
| **Comp 3** | 0.91246539 | 0.33138300 | 0.1304 | 0.7846 |
| **Comp 4** | 0.58108239 | 0.16606832 | 0.0830 | 0.8676 |
| **Comp 5** | 0.41501407 | 0.12475841 | 0.0593 | 0.9269 |
| **Comp 6** | 0.29025567 | 0.06860670 | 0.0415 | 0.9683 |
| **Comp 7** | 0.22164896 |  | 0.0317 | 1.0000 |
