# Supplementary material for: Two Distinct Chronic Obstructive Pulmonary Disease (COPD) Phenotypes Are Associated with High Risk of Mortality
Source: PLoS One. 2012 Dec 7;7(12):e51048. doi: 10.1371/journal.pone.0051048 (PMC3517611; doi:10.1371/journal.pone.0051048)
Supplement: Table S5 — Principal component analysis of 7 continuous variables in 527 patients: correlation coefficients between variables and components identified by principal component analysis. (DOC) [file pone.0051048.s006.doc]

|  | **Comp 1** | **Comp 2** | **Comp 3** | **Comp 4** | **Comp 5** | **Comp 6** | **Comp 7** |
| --- | --- | --- | --- | --- | --- | --- | --- |
| **Age** | 0.036670 | 0.566219 | -.768372 | -.120745 | 0.229984 | -.113309 | 0.085705 |
| **BMI** | -.237273 | 0.533831 | 0.510853 | -.566531 | 0.008452 | -.045886 | 0.273167 |
| **FEV1 % pred** | -.473372 | -.028445 | -.005772 | 0.490249 | 0.196681 | 0.142322 | 0.689778 |
| **mMRC** | 0.422654 | 0.375358 | 0.162881 | 0.241355 | 0.073640 | 0.767032 | -.043902 |
| **CCQ total** | 0.412372 | 0.253968 | 0.333543 | 0.427144 | 0.351826 | -.589719 | 0.014034 |
| **TGV %pred** | 0.379733 | -.433640 | -.034705 | -.427279 | 0.587666 | 0.108745 | 0.356105 |
| **DLCO % pred** | -.474754 | 0.013213 | 0.098055 | 0.029119 | 0.658633 | 0.129880 | -.559737 |
