# Supplementary material for: Two Distinct Chronic Obstructive Pulmonary Disease (COPD) Phenotypes Are Associated with High Risk of Mortality
Source: PLoS One. 2012 Dec 7;7(12):e51048. doi: 10.1371/journal.pone.0051048 (PMC3517611; doi:10.1371/journal.pone.0051048)
Supplement: Table S6 — Relative contribution of the 17 dimensions identified in the multiple correspondence analyses. (DOC) [file pone.0051048.s007.doc]

|  | **Inertia and Chi-Square Decomposition** | | | | | |
| --- | --- | --- | --- | --- | --- | --- |
|  | **Singular Value** | **Principal Inertia** | **Chi-Square** | **Percent** | **Cumulative Percent** | 4    8   12   16   20    ----+----+----+----+----+--- |
| **Dim 1** | 0.60734 | 0.36886 | 2138.3 | 16.91 | 16.91 | ********************* |
| **Dim 2** | 0.57922 | 0.33550 | 1944.9 | 15.38 | 32.28 | ******************* |
| **Dim 3** | 0.54425 | 0.29621 | 1717.1 | 13.58 | 45.86 | ***************** |
| **Dim 4** | 0.37564 | 0.14110 | 818.0 | 6.47 | 52.33 | ******** |
| **Dim 5** | 0.34437 | 0.11859 | 687.5 | 5.44 | 57.76 | ******* |
| **Dim 6** | 0.33102 | 0.10958 | 635.2 | 5.02 | 62.78 | ****** |
| **Dim 7** | 0.31590 | 0.09979 | 578.5 | 4.57 | 67.36 | ****** |
| **Dim 8** | 0.31514 | 0.09931 | 575.7 | 4.55 | 71.91 | ****** |
| **Dim 9** | 0.30521 | 0.09315 | 540.0 | 4.27 | 76.18 | ***** |
| **Dim 10** | 0.29803 | 0.08882 | 514.9 | 4.07 | 80.25 | ***** |
| **Dim 11** | 0.27845 | 0.07753 | 449.5 | 3.55 | 83.80 | **** |
| **Dim 12** | 0.27377 | 0.07495 | 434.5 | 3.44 | 87.24 | **** |
| **Dim 13** | 0.26587 | 0.07069 | 409.8 | 3.24 | 90.48 | **** |
| **Dim 14** | 0.25524 | 0.06515 | 377.7 | 2.99 | 93.47 | **** |
| **Dim 15** | 0.25088 | 0.06294 | 364.9 | 2.88 | 96.35 | **** |
| **Dim 16** | 0.21074 | 0.04441 | 257.5 | 2.04 | 98.39 | *** |
| **Dim 17** | 0.18769 | 0.03523 | 204.2 | 1.61 | 100.00 | ** |
|  | Total | 2.18182 | 12648.0 | 100.00 |  |  |
|  | **Degrees of Freedom = 17884** | | | | | |
