# Supplementary material for: Two Distinct Chronic Obstructive Pulmonary Disease (COPD) Phenotypes Are Associated with High Risk of Mortality
Source: PLoS One. 2012 Dec 7;7(12):e51048. doi: 10.1371/journal.pone.0051048 (PMC3517611; doi:10.1371/journal.pone.0051048)
Supplement: Table S7 — Correlations of the original categorical variables with the 17 dimensions derived from the multiple correspondence analyses. (DOC) [file pone.0051048.s008.doc]

| **Partial Contributions to Inertia for the Column Points** | | | | | | | | | | | | | | | | | |
| --- | --- | --- | --- | --- | --- | --- | --- | --- | --- | --- | --- | --- | --- | --- | --- | --- | --- |
|  | **Dim1** | **Dim2** | **Dim3** | **Dim4** | **Dim5** | **Dim6** | **Dim7** | **Dim8** | **Dim9** | **Dim10** | **Dim11** | **Dim12** | **Dim13** | **Dim14** | **Dim15** | **Dim16** | **Dim17** |
| **IHD yes** | 0.0010 | 0.0015 | 0.0062 | 0.0569 | 0.2021 | 0.0000 | 0.0120 | 0.0003 | 0.0037 | 0.0537 | 0.0385 | 0.0623 | 0.0663 | 0.0407 | 0.2194 | 0.0070 | 0.0003 |
| **IHD no** | 0.0003 | 0.0005 | 0.0018 | 0.0168 | 0.0596 | 0.0000 | 0.0035 | 0.0001 | 0.0011 | 0.0158 | 0.0114 | 0.0184 | 0.0195 | 0.0120 | 0.0647 | 0.0021 | 0.0001 |
| **Diabetes yes** | 0.0003 | 0.0002 | 0.0005 | 0.0814 | 0.0624 | 0.0008 | 0.3597 | 0.0045 | 0.0003 | 0.0006 | 0.0143 | 0.0185 | 0.0000 | 0.2769 | 0.0243 | 0.0194 | 0.0011 |
| **Diabetes unknown** | **0.2328** | 0.0124 | 0.0017 | 0.0001 | 0.0000 | 0.0003 | 0.0003 | 0.0000 | 0.0000 | 0.0003 | 0.0001 | 0.0001 | 0.0003 | 0.0003 | 0.0004 | 0.0004 | 0.0000 |
| **Diabetes no** | 0.0003 | 0.0001 | 0.0001 | 0.0128 | 0.0097 | 0.0001 | 0.0565 | 0.0007 | 0.0000 | 0.0001 | 0.0022 | 0.0028 | 0.0000 | 0.0429 | 0.0039 | 0.0029 | 0.0002 |
| **PAD no** | 0.0036 | 0.0148 | 0.0376 | 0.0002 | 0.0391 | 0.0009 | 0.0475 | 0.0090 | 0.0024 | 0.0002 | 0.0586 | 0.0121 | 0.0031 | 0.0149 | 0.0096 | 0.0197 | 0.0010 |
| **PAD yes** | 0.0003 | 0.0004 | 0.0140 | 0.0285 | 0.2020 | 0.0007 | 0.2097 | 0.0002 | 0.0013 | 0.0009 | 0.1362 | 0.0104 | 0.0002 | 0.0288 | 0.2107 | 0.0218 | 0.0058 |
| **PAD unknown** | 0.0093 | 0.0347 | 0.1223 | 0.0151 | 0.0002 | 0.0034 | 0.0013 | 0.0208 | 0.0085 | 0.0000 | 0.0156 | 0.0093 | 0.0051 | 0.0053 | 0.0186 | 0.0843 | 0.0000 |
| **Stroke no** | 0.0003 | 0.0002 | 0.0001 | 0.0000 | 0.0000 | 0.0001 | 0.0015 | 0.0158 | 0.0005 | 0.0078 | 0.0068 | 0.0000 | 0.0020 | 0.0000 | 0.0010 | 0.0002 | 0.0002 |
| **Stroke unknown** | **0.2328** | 0.0124 | 0.0017 | 0.0001 | 0.0000 | 0.0003 | 0.0003 | 0.0000 | 0.0000 | 0.0003 | 0.0001 | 0.0001 | 0.0003 | 0.0003 | 0.0004 | 0.0004 | 0.0000 |
| **Stroke yes** | 0.0004 | 0.0030 | 0.0017 | 0.0010 | 0.0010 | 0.0035 | 0.0393 | 0.4198 | 0.0135 | 0.2109 | 0.1791 | 0.0014 | 0.0507 | 0.0001 | 0.0273 | 0.0049 | 0.0063 |
| **Osteop. no** | 0.0001 | 0.0006 | 0.0025 | 0.0114 | 0.0152 | 0.0208 | 0.0016 | 0.0001 | 0.0151 | 0.0001 | 0.0025 | 0.0594 | 0.0429 | 0.0017 | 0.0013 | 0.0015 | 0.0004 |
| **Osteop yes** | 0.0006 | 0.0016 | 0.0108 | 0.0525 | 0.0705 | 0.0981 | 0.0080 | 0.0005 | 0.0705 | 0.0005 | 0.0117 | 0.2775 | 0.1984 | 0.0074 | 0.0058 | 0.0072 | 0.0018 |
| **Osteop unknown** | **0.2328** | 0.0124 | 0.0017 | 0.0001 | 0.0000 | 0.0003 | 0.0003 | 0.0000 | 0.0000 | 0.0003 | 0.0001 | 0.0001 | 0.0003 | 0.0003 | 0.0004 | 0.0004 | 0.0000 |
| **Muscle W yes** | 0.0016 | 0.0071 | 0.0174 | 0.0705 | 0.0274 | 0.1304 | 0.0254 | 0.0022 | 0.0015 | 0.0331 | 0.1099 | 0.0027 | 0.1842 | 0.0161 | 0.0001 | 0.0399 | 0.0007 |
| **Muscle W unknown** | 0.0093 | 0.0347 | 0.1223 | 0.0151 | 0.0002 | 0.0034 | 0.0013 | 0.0208 | 0.0085 | 0.0000 | 0.0156 | 0.0093 | 0.0051 | 0.0053 | 0.0186 | 0.0843 | 0.0000 |

| **Partial Contributions to Inertia for the Column Points** | | | | | | | | | | | | | | | | | |
| --- | --- | --- | --- | --- | --- | --- | --- | --- | --- | --- | --- | --- | --- | --- | --- | --- | --- |
|  | **Dim1** | **Dim2** | **Dim3** | **Dim4** | **Dim5** | **Dim6** | **Dim7** | **Dim8** | **Dim9** | **Dim10** | **Dim11** | **Dim12** | **Dim13** | **Dim14** | **Dim15** | **Dim16** | **Dim17** |
| **Muscle W no** | 0.0022 | 0.0074 | 0.0331 | 0.0121 | 0.0146 | 0.1117 | 0.0093 | 0.0066 | 0.0114 | 0.0208 | 0.1334 | 0.0144 | 0.0793 | 0.0017 | 0.0110 | 0.0063 | 0.0004 |
| **Anemia yes** | 0.0003 | 0.0023 | 0.0002 | 0.0427 | 0.0627 | 0.2405 | 0.0094 | 0.0169 | 0.0074 | 0.0572 | 0.0079 | 0.2641 | 0.1397 | 0.0038 | 0.0497 | 0.0028 | 0.0069 |
| **Anemia unknown** | **0.2328** | 0.0124 | 0.0017 | 0.0001 | 0.0000 | 0.0003 | 0.0003 | 0.0000 | 0.0000 | 0.0003 | 0.0001 | 0.0001 | 0.0003 | 0.0003 | 0.0004 | 0.0004 | 0.0000 |
| **Anemia no** | 0.0003 | 0.0004 | 0.0000 | 0.0040 | 0.0058 | 0.0223 | 0.0008 | 0.0016 | 0.0007 | 0.0055 | 0.0007 | 0.0249 | 0.0132 | 0.0003 | 0.0045 | 0.0002 | 0.0006 |
| **Emph yes** | 0.0000 | 0.0001 | 0.0632 | 0.0581 | 0.0012 | 0.0099 | 0.0000 | 0.0015 | 0.0007 | 0.0012 | 0.0005 | 0.0036 | 0.0007 | 0.0051 | 0.0015 | 0.0104 | 0.0074 |
| **Emph unknown** | 0.0065 | **0.1901** | 0.0419 | 0.0000 | 0.0016 | 0.0001 | 0.0012 | 0.0001 | 0.0001 | 0.0000 | 0.0022 | 0.0000 | 0.0000 | 0.0039 | 0.0004 | 0.0000 | **0.2338** |
| **Emph no** | 0.0014 | 0.0284 | 0.0946 | 0.1332 | 0.0044 | 0.0237 | 0.0002 | 0.0031 | 0.0019 | 0.0027 | 0.0024 | 0.0085 | 0.0017 | 0.0167 | 0.0042 | 0.0243 | 0.0016 |
| **Airway 0** | 0.0045 | 0.0135 | 0.0347 | 0.0317 | 0.0003 | 0.0239 | 0.0164 | 0.0662 | 0.0014 | 0.0030 | 0.0351 | 0.0363 | 0.0088 | 0.1367 | 0.0231 | 0.1825 | 0.0001 |
| **Airway 1** | 0.0005 | 0.0005 | 0.0173 | 0.0008 | 0.0063 | 0.0005 | 0.0420 | 0.1176 | 0.1197 | 0.0391 | 0.0769 | 0.0636 | 0.0122 | 0.0508 | 0.0006 | 0.0184 | 0.0093 |
| **Airway 2** | 0.0009 | 0.0005 | 0.0248 | 0.0531 | 0.0072 | 0.0315 | 0.0164 | 0.0329 | 0.3480 | 0.0496 | 0.0207 | 0.0092 | 0.0011 | 0.0301 | 0.0300 | 0.1600 | 0.0048 |
| **Airway unknown** | 0.0065 | **0.1880** | 0.0450 | 0.0010 | 0.0007 | 0.0009 | 0.0000 | 0.0050 | 0.0001 | 0.0000 | 0.0005 | 0.0011 | 0.0000 | 0.0022 | 0.0008 | 0.0013 | **0.2318** |
| **Bronch 0** | 0.0011 | 0.0096 | 0.0040 | 0.0067 | 0.0065 | 0.0144 | 0.0008 | 0.0536 | 0.0416 | 0.0004 | 0.0119 | 0.0015 | 0.0212 | 0.0081 | 0.0368 | 0.0323 | 0.0076 |
| **Bronch 1** | 0.0009 | 0.0003 | 0.0345 | 0.0237 | 0.0174 | 0.0395 | 0.0023 | 0.1440 | 0.1292 | 0.0012 | 0.0395 | 0.0032 | 0.0645 | 0.0303 | 0.1195 | 0.1067 | 0.0004 |
| **Bronch unknown** | 0.0065 | **0.1880** | 0.0450 | 0.0010 | 0.0007 | 0.0009 | 0.0000 | 0.0050 | 0.0001 | 0.0000 | 0.0005 | 0.0011 | 0.0000 | 0.0022 | 0.0008 | 0.0013 | **0.2318** |
| **ALV_0** | 0.0014 | 0.0284 | 0.0946 | 0.1332 | 0.0044 | 0.0237 | 0.0002 | 0.0031 | 0.0019 | 0.0027 | 0.0024 | 0.0085 | 0.0017 | 0.0167 | 0.0042 | 0.0243 | 0.0016 |
| **ALV_1** | 0.0001 | 0.0011 | 0.0040 | 0.1223 | 0.0715 | 0.0773 | 0.0528 | 0.0143 | 0.1098 | 0.0121 | 0.0058 | 0.0243 | 0.0135 | 0.0731 | 0.0321 | 0.0356 | 0.0003 |
| **ALV_2** | 0.0009 | 0.0002 | 0.0329 | 0.0087 | 0.0022 | 0.0884 | 0.0001 | 0.0250 | 0.0981 | 0.3175 | 0.0461 | 0.0122 | 0.0022 | 0.0056 | 0.0734 | 0.0216 | 0.0047 |
| **ALV_3** | 0.0009 | 0.0021 | 0.0440 | 0.0052 | 0.1012 | 0.0271 | 0.0782 | 0.0090 | 0.0006 | 0.1620 | 0.0090 | 0.0389 | 0.0612 | 0.1557 | 0.0000 | 0.0752 | 0.0050 |
| **ALV_ unknown** | 0.0065 | **0.1901** | 0.0419 | 0.0000 | 0.0016 | 0.0001 | 0.0012 | 0.0001 | 0.0001 | 0.0000 | 0.0022 | 0.0000 | 0.0000 | 0.0039 | 0.0004 | 0.0000 | **0.2338** |

Abbreviations: IHD: ischemic heart disease; PAD: peripheral arterial disease; Osteop: osteoporosis; Muscle W: muscle weakness; Emph: emphysema; Airway: airway thickening; Bronch: bronchiectasis; ALV: alveolar destruction.
