# Supplementary material for: Two Distinct Chronic Obstructive Pulmonary Disease (COPD) Phenotypes Are Associated with High Risk of Mortality
Source: PLoS One. 2012 Dec 7;7(12):e51048. doi: 10.1371/journal.pone.0051048 (PMC3517611; doi:10.1371/journal.pone.0051048)
Supplement: Table S8 — Comparison of included vs. excluded subjects from the cluster analysis. (DOC) [file pone.0051048.s009.doc]

|  | **Included subjects** | **Excluded subjects** | **P values** |
| --- | --- | --- | --- |
|  | **n=527** | **n=122** |  |
| **Male, %** | 78 | 57 | *P*<0.001 |
| **Age, yrs.** | 65 [60-72] | 57 [54-62] | *P*<0.001 |
| **FEV_1_, % predicted** | 55 [36-78] | 24 [19-31] | *P*<0.001 |
| **BMI, kg/ m^2^** | 24.8 [21.8-27.7] | 21.5 [18.2-24.8] | *P*<0.001 |
| **Follow up time, yrs** | 2.4 [1.9-2.9] | 5.3 [3.7-7.4] | *P*<0.001 |
